# Physicians’ Attitudes and Practices Regarding Drug Allergy Management: A Cross-Sectional Study

**DOI:** 10.7759/cureus.82012

**Published:** 2025-04-10

**Authors:** Elias A Alraqibah

**Affiliations:** 1 Department of Medicine, College of Medicine, Qassim University, Buraydah, SAU

**Keywords:** anaphylaxis, drug allergy, drug hypersensitivity, medical education, physician practice patterns, saudi arabia

## Abstract

Introduction

Drug-induced anaphylaxis is a life-threatening hypersensitivity reaction requiring prompt recognition and management by physicians. Although clinically significant, studies have revealed gaps in physicians' attitudes and practices regarding drug-induced anaphylaxis, potentially affecting patient safety. This study assessed physicians' attitudes and practices related to drug-induced allergies.

Methods

A cross-sectional study was conducted among 167 physicians across four hospitals in the Qassim region using a validated structured questionnaire. The tool assessed three domains: demographic characteristics, attitudes toward drug allergy management, and clinical practices. Data were analyzed using descriptive statistics with IBM SPSS Statistics software, version 26 (IBM Corp., Armonk, NY).

Results

A total of 167 physicians were included in the study. Attitudes toward drug allergy management were largely positive, with 91.6% (n=153) recognizing the need for advanced training. However, practice patterns varied, with only 50.3% (n=84) consistently recognizing and managing drug hypersensitivity reactions. The availability and routine use of drug allergy testing was reported by 47.3% (n=79), yet accurate interpretation remained low (31.9%, n=53). Although 65.7% (n=110) routinely obtained allergy histories, proper skin test controls were performed by only 20.1% (n=34).

Conclusion

While physicians exhibited positive attitudes toward drug allergy management, inconsistencies in clinical practice were evident. Deficiencies in diagnostic awareness, reliance on inaccurate testing methods, and variable adherence to management guidelines underscore the need for targeted educational interventions.

## Introduction

Allergies to drug administration represent a significant challenge in clinical practice that can lead to several adverse drug reactions, delays in treatment, increased healthcare costs, and increased morbidity and mortality rates [1,2]. Drug allergy is defined as an immunologically mediated hypersensitivity reaction to certain medications, ranging from mild cutaneous symptoms to severe, life-threatening anaphylaxis [3,4]. Accurate diagnosis and prompt management are crucial for preventing unnecessary drug avoidance while ensuring optimal patient care [5,6]. However, research indicates that mislabeling of drug allergies, particularly to beta-lactam antibiotics, frequently results in suboptimal treatment choices and contributes to antibiotic resistance [7].

Physicians play a critical role in identifying, documenting, and managing drug allergies [8]. Their knowledge, attitudes, and practices (KAP) directly influence patient safety and treatment efficacy [9,10]. Across several nations, studies have shown variations in doctors' knowledge and adherence to drug allergy treatment protocols [11,12]. These challenges may be exacerbated by inconsistent allergy documentation practices and limited guidance on proper allergy evaluation procedures. Furthermore, physicians demonstrate varying approaches to allergy testing, risk stratification, and removal of incorrect allergy labels, potentially impacting clinical outcomes [12].

Despite growing recognition of these issues, data on drug allergy management practices among physicians in Saudi Arabia remain limited. This knowledge gap is particularly concerning given the rising prevalence of allergic diseases and increasing complexity of modern pharmacotherapy. This study aimed to evaluate physicians' attitudes and clinical practices regarding drug allergy management in the Qassim region of Saudi Arabia. By identifying existing gaps and challenges, this research seeks to inform targeted educational interventions, promote evidence-based allergy management, and ultimately enhance patient safety.

## Materials and methods

Study design and setting

This cross-sectional study was conducted among physicians working in four major hospitals in the Qassim region of Saudi Arabia, namely, King Fahad Specialist Hospital, Buraydah Central Hospital, King Saud Hospital, and Maternity and Children Hospital. The study aimed to evaluate physicians’ attitudes and practices regarding drug allergy management. Data collection occurred between 2023 and 2024.

Study population and sampling

The target population included physicians actively involved in patient care across secondary and tertiary hospitals. Administrative staff, temporary leave personnel, and those without direct patient contact were excluded. The minimum sample size (n=200) was determined using the finite population formula (N=750 physicians, 90% CI, 5% margin of error, 50% expected proportion). Of 320 physicians contacted through stratified random sampling, 167 completed the survey, yielding a 52.2% response rate.

Data collection tool

A structured, self-administered questionnaire (Appendix A) was adapted from a validated tool used in a prior study by Wang et al. [13]. The questionnaire comprised 19 items divided into three sections. The first section collected demographic information, including age, gender, education level, professional title, and hospital level. The second section assessed attitudes toward drug allergy, focusing on perceptions of its importance, the need for training, and self-rated knowledge. The third section evaluated practices related to drug allergy management, such as adherence to testing protocols, history-taking, and institutional support. The questionnaire was distributed both in person and via Google Forms (Google LLC, Mountain View, CA), and participants provided informed consent before participation.

Data analysis

Data were analyzed using IBM SPSS Statistics software, version 26 (IBM Corp., Armonk, NY). Descriptive statistics (means, standard deviations, frequencies, and percentages) summarized demographic and categorical variables.

Ethical considerations

Ethical approval for this study was obtained from the Qassim Regional Research Ethics Committee (Approval number: 607/44/10971). The study strictly adhered to the principles outlined in the Declaration of Helsinki and followed Saudi Arabia’s national ethical guidelines for biomedical research. Written informed consent was obtained from all participants prior to enrollment, with clear explanations of the study’s purpose, procedures, and their right to withdraw without consequence. To ensure confidentiality, no personally identifiable information was collected, and all data were anonymized during analysis. Electronic responses were stored on password-protected servers, while paper forms were kept in locked cabinets accessible only to the research team. Participation was entirely voluntary, and no incentives were offered to avoid coercion.

## Results

Demographics of participants

A total of 167 physicians participated in the study, the majority being male (81.4%, n=136) and holding a bachelor's degree (54.5%, n=91). Participants included residents or fellows (46.1%, n=77), consultants or specialists (41.3%, n=69), and interns (12.6%, n=21). Most worked in tertiary hospitals (80.2%, n=134), while a smaller proportion were in secondary hospitals (19.8%, n=33) (Table 1).

**Table 1:** Demographic characteristics of the participating physicians (N=167)

Demographic characteristics		Count (n)	Percentage (%)
Gender	Male	136	81.40%
	Female	31	18.60%
Education	Bachelor	91	54.50%
	Masters/Board-certified and above	76	45.50%
Title	Intern	21	12.60%
	Resident/Fellow	77	46.10%
	Specialist/Consultant	69	41.30%
Level of hospital	Tertiary hospital	134	80.20%
	Secondary hospital	33	19.80%

Attitudes toward drug allergy management

The majority of physicians demonstrated positive attitudes toward drug allergy management. Notably, 91.6% (n=153) agreed or strongly agreed that healthcare practitioners should receive advanced training in drug hypersensitivity reactions. Additionally, 61.6% (n=103) believed that in vivo or in vitro drug testing is important before administration, while 29.3% (n=49) were uncertain, and 9.0% (n=15) disagreed. Regarding self-perceived knowledge, 40.1% (n=67) of physicians expressed satisfaction with their understanding of drug hypersensitivity reactions, while 24.6% (n=41) were uncertain, and 22.8% (n=38) reported dissatisfaction. A significant proportion (91.6%, n=153) agreed or strongly agreed that drug allergies adversely affect patients' quality of life. However, only 45.5% (n=76) believed that drug allergies frequently occurred in their daily practice, with 24.6% (n=41) remaining uncertain. In terms of case frequency, 46.7% (n=78) encountered fewer than one drug allergy case per month, while 38.3% (n=64) reported seeing one to five cases monthly (Table 2).

**Table 2:** Physicians' attitudes toward drug allergy management and perceived clinical impact (N=167)

Questions		Count (n)	Percentage (%)
Do you think healthcare practitioners (HCPs) should receive advanced knowledge and training in drug hypersensitivity reactions (DHRs)?	Strongly disagree	3	1.8%
	Disagree	2	1.2%
	Uncertain	9	5.4%
	Agree	64	38.3%
	Strongly agree	89	53.3%
Do you think an in vivo or in vitro test of the drug is very important before‎ administration?	Strongly disagree	2	1.2%
	Disagree	13	7.8%
	Uncertain	49	29.3%
	Agree	56	33.5%
	Strongly agree	47	28.1%
Are you satisfied with your knowledge of DHRs?	Strongly disagree	6	3.6%
	Disagree	32	19.2%
	Uncertain	41	24.6%
	Agree	67	40.1%
	Strongly agree	21	12.6%
Do you think drug allergies have an adverse impact on a patient’s quality of life?	Strongly disagree	5	3.0%
	Disagree	3	1.8%
	Uncertain	6	3.6%
	Agree	71	42.5%
	Strongly agree	82	49.1%
Do you think drug allergies always occur in your daily practice?	Strongly disagree	12	7.2%
	Disagree	38	22.8%
	Uncertain	41	24.6%
	Agree	59	35.3%
	Strongly agree	17	10.2%
On average, how many cases of drug allergies do you see in your daily practice monthly?	Less than 1 case/month	78	46.7%
	1 to 5 cases	64	38.3%
	More than 5 cases	8	4.8%
	Never	17	10.2%

Clinical practices and institutional resources

Clinical practices showed inconsistencies. While 65.7% (n=110) consistently obtained allergy histories before prescribing medications, only 31.9% (n=53) accurately evaluated drug skin test results, and just 20.1% (n=34) performed proper positive and negative controls. Access to drug allergy testing also varied; 47.3% (n=79) reported it was available upon request, whereas 6.1% (n=10) indicated it was neither available nor accessible (Table 3). Physicians also reported challenges in managing drug hypersensitivity reactions. While 50.3% (n=84) always recognized and addressed these reactions promptly, access to specialized consultations was inconsistent; 39.5% (n=66) relied on internal medicine specialists, 31.1% (n=52) consulted immunologists, and 13.8% (n=23) had no access to allergy specialists.

**Table 3:** Clinical practices and institutional resources for drug allergy management among study participants (N=167) DHR: drug hypersensitivity reactions

Questions		Count (n)	Percentage (%)
At your institute, a drug allergy test is available and/or performed.	Available and performed once requested	78	47.30%
	Available, but not routinely performed	40	24.20%
	Available, but only for certain specialties	19	11.50%
	Not available, but can be requested	18	10.90%
	Not available and cannot be requested	10	6.10%
Do you take the patient’s (any) allergy history before drug prescribing/administration or upon admission?	Never	2	1.20%
	Rarely	6	3.60%
	Sometimes	18	10.80%
	Often	31	18.70%
	Always	109	65.70%
Do you evaluate the drug skin test result timely and accurately (if available)?	Never	15	9.20%
	Rarely	19	11.70%
	Sometimes	38	23.30%
	Often	39	23.90%
	Always	52	31.90%
Do you perform positive control and negative control during drug skin tests?	Never	45	27.40%
	Rarely	27	16.50%
	Sometimes	40	24.40%
	Often	19	11.60%
	Always	33	20.10%
Do you recognize and manage DHRs in a timely manner when they occur?	Never	14	8.40%
	Rarely	10	6.00%
	Sometimes	27	16.20%
	Often	32	19.20%
	Always	84	50.30%
At your institute, is there an available team, specialty, or doctor that can be consulted in case of drug allergy patients?	Immunologist	52	31.10%
	Internal medicine	66	39.50%
	Allergic expert	26	15.60%
	None	23	13.80%
At your institute, is there an available protocol or a guideline that can be referred to in case of drug allergy patients?	Yes, it is available and widely distributed	74	44.30%
	Yes, it is available, but not distributed	48	28.70%
	Yes, but not available or outdated	16	9.60%
	None	29	17.40%
Do you participate in continuous medical education regarding drug allergy?	Never	13	7.80%
	Rarely	36	21.60%
	Sometimes	49	29.30%
	Often	41	24.60%
	Always	28	16.80%
At your institute, do you find the required information about a patient's allergy?	Never	7	4.20%
	Rarely	13	7.80%
	Sometimes	41	24.70%
	Often	48	28.90%
	Always	57	34.30%

Guideline availability varied, with 44.3% (n=74) reporting their institutions had widely distributed protocols, while 17.4% (n=29) had no guidelines available. Participation in continuous medical education (CME) was inconsistent, as only 16.8% (n=28) regularly attended allergy-related training, while 29.4% (n=49) rarely or never did. Access to allergy-related patient information was also inconsistent, with only 34.3% (n=57) always finding necessary records (Table 3).

## Discussion

This study evaluated physicians' attitudes and practices regarding drug allergy management in secondary and tertiary hospitals in the Qassim region of Saudi Arabia. The findings revealed a generally positive attitude among physicians, yet significant gaps in clinical practices were identified. These results align with previous studies conducted in Saudi Arabia and other regions, highlighting both progress and persistent challenges in drug allergy management [11,14]. However, a study by Ahmad et al. conducted among pharmacists in India reported a negative attitude toward drug allergy management [15]. A majority of participants (91.6%) agreed or strongly agreed that healthcare practitioners should receive advanced training in drug hypersensitivity reactions, reflecting a willingness to improve competence through continued education, which is higher than reported in the study from Riyadh (42 %) [14] and similar to what was reported in the previous study conducted in China [13]. Additionally, 61.6% acknowledged the importance of in vivo or in vitro testing before drug administration, reinforcing the role of standardized allergy testing in preventing adverse drug reactions [16,17].

Moreover, 52.7% of physicians expressed satisfaction with their knowledge of drug hypersensitivity reactions, indicating a potential overestimation of competency. This is higher than what was reported in the study from Riyadh, where 33 % of the participants were satisfied with their knowledge of drug hypersensitivity reactions [14]. China’s study reported that only one-third were confident in their knowledge [13]. The strong agreement (91.6%) that drug allergies negatively impact patients’ quality of life aligns with existing literature emphasizing the psychological and medical burden of drug hypersensitivity reactions [18-20]. This is similar to a study from Malaysia, which reported that 93.1 % of the participants reiterated that drug allergy could adversely impact the patient’s quality of life [11].

Despite positive attitudes, physicians' actual practices varied. This is consistent with the results of Riyadh’s study, which also reported that the participants' positive attitudes did not translate into clinical practice [14]. While 47.3% reported that drug allergy tests were available and performed upon request at their institutions, 24.2% noted availability without routine performance, and 6.1% indicated unavailability. These findings suggest that institutional limitations may contribute to inconsistent implementation of best practices [21].

Additionally, 65.7% of participants consistently took patients' allergy histories before prescribing or administering drugs, highlighting a fundamental but sometimes overlooked aspect of safe prescribing [14]. This is significantly higher than reported in the Riyadh study, where only 22 % of the participants reported taking a history of drug allergies [14]; however, this is lower than reported in a previous study conducted in China, which reported that 90% of the respondents would take patients’ allergic history before drug administration [13]. However, only 31.9% consistently evaluated drug skin test results accurately and promptly, and just 20.1% always performed both positive and negative controls during drug skin tests. This variability in adherence to best practices suggests a need for standardized training programs to ensure uniformity in drug allergy testing and interpretation [22].

Furthermore, only 50.3% of physicians consistently recognized and managed drug hypersensitivity reactions promptly. This finding is concerning, as delayed recognition and intervention can lead to severe patient outcomes, including anaphylactic shock and death [23]. Institutions should prioritize thorough training courses covering case-based learning, simulation drills, and standardized anaphylaxis diagnosis and treatment procedures. Moreover, guaranteeing drug allergy testing is necessary to enhance clinical results and stress the need for thorough patient allergy histories. The involvement of various medical professionals allows for a comprehensive review of their attitudes and practices. This study's primary advantage is its emphasis on physician readiness to manage drug-induced allergies.

While this study provides valuable insights into physicians’ attitudes and practices regarding drug allergy management, several limitations must be acknowledged. First, the reliance on self-reported data through questionnaires may introduce response bias, as participants might overestimate their knowledge or adherence to best practices. Second, the cross-sectional design limits our ability to establish causal relationships between attitudes and practices over time. Third, the study was conducted in four major hospitals in the Qassim region, which may not fully represent the practices of physicians in smaller healthcare facilities or other regions of Saudi Arabia. Additionally, the sample size (n = 167), though adequate for preliminary analysis, could be expanded in future studies to enhance generalizability. Moreover, the study did not assess the actual clinical outcomes of drug allergy management (e.g., patient adverse events), which would provide a more objective measure of practice efficacy. Moreover, our 52.2% response rate may have introduced non-response bias if physicians with differing attitudes or practices toward drug allergy management were systematically less likely to participate. Although the questionnaire was adapted from a validated tool, we did not perform a formal psychometric evaluation of the survey instrument in our specific study population, which could affect the reliability and validity of the measured constructs. Finally, the analysis was restricted to descriptive statistics; inferential analyses could have provided deeper insights into the factors influencing drug allergy management.

Despite these limitations, the study has notable strengths, including its rigorous ethical oversight, direct relevance to clinical practice, and its focus on a critical yet understudied aspect of patient safety in the Saudi context. The findings lay the groundwork for future research employing longitudinal designs, multi-center collaborations, and mixed-methods approaches to validate and extend our observations.

## Conclusions

This study highlights the generally positive attitudes of physicians in the Qassim region of Saudi Arabia toward drug allergy management, with a strong recognition of the need for advanced training. However, notable inconsistencies in clinical practice were observed, including variability in the recognition and management of drug hypersensitivity reactions, limited use and interpretation of drug allergy testing, and inadequate adherence to guideline-based diagnostic approaches. These findings underscore the need for targeted educational interventions and standardized institutional protocols to enhance physicians' knowledge and clinical practices. Strengthening training programs and promoting evidence-based allergy management strategies will ultimately improve patient safety and reduce the risks associated with drug hypersensitivity reactions. Given the study's descriptive nature, these observations should be interpreted as indicative rather than conclusive regarding their impact on patient outcomes. We recommend implementing mandatory CME programs focused on drug hypersensitivity, including case-based learning and simulation training for anaphylaxis management. Hospitals should adopt standardized institutional protocols to ensure uniform diagnostic and treatment approaches. Integrating electronic health record (EHR) alerts for allergy documentation and specialist referrals could further reduce errors. Additionally, improving access to drug allergy testing, particularly in tertiary centers, and establishing multidisciplinary allergy stewardship programs involving pharmacists and immunologists would enhance diagnostic accuracy and reduce unnecessary drug avoidance. Regular audits of allergy documentation and testing practices should be conducted to monitor compliance and identify areas for improvement. Future research should explore the impact of structured educational programs on improving drug allergy diagnosis and management in healthcare settings.

## Appendices

Appendix A 

**Table 4:** Study questionnaire

Variable	Options
Gender	Male
	Female
Education	Bachelor
	Masters/Board-certified and above
Title	Intern
	Resident/Fellow
	Specialist/Consultant
Level of hospital	Tertiary hospital
	Secondary hospital
Attitude	
Question	Response options
1. Do you think healthcare practitioners (HCPs) should receive advanced knowledge and training of drug hypersensitivity reactions (DHRs)?	a) Strongly agree
	b) Agree
	c) Uncertain
	d) Disagree
	e) Strongly disagree
2. Do you think in vivo or in vitro testing of drugs is very important before administration?	a) Strongly agree
	b) Agree
	c) Uncertain
	d) Disagree
	e) Strongly disagree
3. Are you satisfied with your knowledge of DHRs?	a) Strongly agree
	b) Agree
	c) Uncertain
	d) Disagree
	e) Strongly disagree
4. Do you think drug allergy has an adverse impact on a patient’s quality of life?	a) Strongly agree
	b) Agree
	c) Uncertain
	d) Disagree
	e) Strongly disagree
5. Do you think drug allergy always occurs in your daily practice?	a) Strongly agree
	b) Agree
	c) Uncertain
	d) Disagree
	e) Strongly disagree
6. On average, how many cases of drug allergy do you see in your daily practice, monthly?	a) Less than 1 case/month
	b) 1 to 5 cases
	c) More than 5 cases
	d) Never
Practice	
Question	Response Options
1. At your institute, is drug allergy testing available and/or performed?	a) Available and performed once requested
	b) Available, but not routinely performed
	c) Available, but only for certain specialties
	d) Not available, but can be requested
	e) Not available, and can’t be requested
2. Do you take the patient’s (any) allergy history before drug prescribing/administration or upon admission?	a) Always
	b) Often
	c) Sometimes
	d) Rarely
	e) Never
3. Do you evaluate the drug skin test result timely and accurately (if available)?	a) Always
	b) Often
	c) Sometimes
	d) Rarely
	e) Never
4. Do you perform positive and negative controls during drug skin testing?	a) Always
	b) Often
	c) Sometimes
	d) Rarely
	e) Never
5. Do you recognize and manage DHRs in a timely manner when they occur?	a) Always
	b) Often
	c) Sometimes
	d) Rarely
	e) Never
6. At your institute, is there an available team, specialty, or doctor that can be consulted in case of drug allergy patients?	a) Immunologist
	b) Internal medicine
	c) Allergy expert
	d) None
7. At your institute, is there an available protocol or guideline that can be referred to in case of drug allergy patients?	a) Yes, available and widely distributed
	b) Yes, available but not distributed
	c) Yes, but not available or outdated
	d) None
8. Do you participate in continuous medical education regarding drug allergy?	a) Always
	b) Often
	c) Sometimes
	d) Rarely
	e) Never
9. At your institute, do you find the required information on a patient's allergy?	a) Always
	b) Often
	c) Sometimes
	d) Rarely
	e) Never
